# Scent Lure Effect on Camera-Trap Based Leopard Density Estimates

**DOI:** 10.1371/journal.pone.0151033

**Published:** 2016-04-06

**Authors:** Alexander Richard Braczkowski, Guy Andrew Balme, Amy Dickman, Julien Fattebert, Paul Johnson, Tristan Dickerson, David Whyte Macdonald, Luke Hunter

**Affiliations:** 1 Wildlife Conservation Research Unit, Department of Zoology, University of Oxford, The Recanati-Kaplan Centre, Tubney House, Tubney, Oxfordshire, United Kingdom; 2 Panthera, 8 West 40th Street,18th Floor, New York, New York, United States of America; 3 Department of Biological Sciences, University of Cape Town, Rondebosch, South Africa; 4 Ruaha Carnivore Project, Iringa, Tanzania; 5 School of Life Sciences, University of Kwazulu-Natal, Westville Campus, Durban, South Africa; University of Bern, SWITZERLAND

## Abstract

Density estimates for large carnivores derived from camera surveys often have wide confidence intervals due to low detection rates. Such estimates are of limited value to authorities, which require precise population estimates to inform conservation strategies. Using lures can potentially increase detection, improving the precision of estimates. However, by altering the spatio-temporal patterning of individuals across the camera array, lures may violate closure, a fundamental assumption of capture-recapture. Here, we test the effect of scent lures on the precision and veracity of density estimates derived from camera-trap surveys of a protected African leopard population. We undertook two surveys (a ‘control’ and ‘treatment’ survey) on Phinda Game Reserve, South Africa. Survey design remained consistent except a scent lure was applied at camera-trap stations during the treatment survey. Lures did not affect the maximum movement distances (p = 0.96) or temporal activity of female (p = 0.12) or male leopards (p = 0.79), and the assumption of geographic closure was met for both surveys (p >0.05). The numbers of photographic captures were also similar for control and treatment surveys (p = 0.90). Accordingly, density estimates were comparable between surveys (although estimates derived using non-spatial methods (7.28–9.28 leopards/100km^2^) were considerably higher than estimates from spatially-explicit methods (3.40–3.65 leopards/100km^2^). The precision of estimates from the control and treatment surveys, were also comparable and this applied to both non-spatial and spatial methods of estimation. Our findings suggest that at least in the context of leopard research in productive habitats, the use of lures is not warranted.

## Introduction

Camera-traps are widely used as a research tool to study cryptic species [1]. In particular, closed capture-recapture (CR) models are often used on camera-trapping data to estimate the population abundance and density of large carnivores [2]. This method has recently been strengthened by incorporating data on spatial information of captured individuals into the CR framework, removing the edge effect typically associated with traditional non-spatial estimators [3], [4]. Importantly, the effective survey area is no longer defined by the inclusion of an ad hoc buffer extending beyond the camera-trap grid [5], but by a homogenous distribution of potential home-range centres, from which density is estimated [3], [6]. Previous non-spatial estimators did not allow for demographic processes like immigration or emigration in their statistical framework in order to interpret N in a meaningful way, and also assumed geographic and demographic closure [1]. Contrastingly new spatial methods relax geographic closure and allow for temporary movement of individuals around the state space [7].

Despite these recent advances, biologists continue to seek ways to improve camera-trap surveys; most notably, their precision given the low detection rates often associated with them [8]. Precise population estimates are required to inform appropriate conservation actions and to monitor the outcomes of management decisions [9]. Increasing the number of recaptures can reduce error but this may also violate the fundamental principles of CR; as sampling periods must be short enough to prevent any demographic processes to occur [10], [11]. Similarly, increasing sampling effort by increasing the number of camera-traps deployed is not always logistically feasible [12]. Increasing the precision of camera-trap studies can be improved by augmenting camera-trap data with ancillary biological information e.g. from faecal DNA [13], [14], but even this may be practically challenging and an inability to age scats or classify those of juveniles may overinflate density estimates. This was shown by Jenecka et al [15] working on snow leopards in the Gobi desert of Mongolia.

An alternative option increasingly used in camera-trap studies is to entice animals to camera-traps by placing an attractant nearby [16]. This may be a scent lure such as a perfume/cologne [17], [18] or a food attractant that is inaccessible to the animal [8]. Baiting is another strategy and entails the use of a food reward such as a carcass or meat [19], [20], [21]. However, the use of attractants can be laborious and expensive, and they are only likely warranted if they increase capture rates significantly [16]. Attractants may also influence the movement of individuals on and off the camera-trap array, potentially violating the assumption of geographic closure [8], but this is relaxed in SCR and individuals may move temporarily around the camera-trap grid [7]. They may also have variable effects on animals depending on their age, sex or resident status. Despite this, attractants have been used in camera-trap studies on species ranging from rodents to large carnivores [19], [20]. The use of lures could also be useful if the total number of animal detections at a sampling site may be used and no sub-division of captures into individual temporal occasions occurs [22].

Attaining a better understanding of the effects of attractants in camera-trap surveys is necessary to determine whether they are an appropriate means of improving the precision of population density estimates.

In this paper we examine how the use of a scent lure affects the behaviour of African leopards *Panthera pardus* during a camera-trap survey, and the precision of the resultant density estimates. We use leopards as a model species as, like many large carnivores, they are of conservation concern as they are sensitive to anthropogenic mortality and have suffered significant range loss in recent decades [23]. Leopards are also important revenue generators for trophy hunting [24] and photo-tourism [25] industries. Therefore, accurate and precise estimates of leopard population density are required to inform conservation and management practices [26], but leopard numbers are difficult to monitor. In this paper we assess (i) whether the use of lures violates the assumptions of geographic closure by examining the spatio-temporal patterning (as defined by the distances animals move between camera-traps, and the timing of photographic captures) of leopard captures in a control (i.e. non-lure) and treatment survey (scent lure), (ii) whether the use of lures significantly increases the number of leopard captures and recaptures, and finally (iii) how precision and density estimates varied between a traditional non-spatial CR density estimation method and two recent spatially-explicit CR approaches.

## Methods

### Ethics statement

We obtained permission to perform leopard research on Phinda Game Reserve from the provincial conservation authority, Ezemvelo Kwazulu-Natal Wildlife (permit number HO/4004/07), and by & Beyond, the management authority on Phinda Game Reserve. Ethical clearance for this research was provided by the University of Kwazulu-Natal Ethics Committee (approval 051/12/Animal).

### Study area

**Fig 1:**
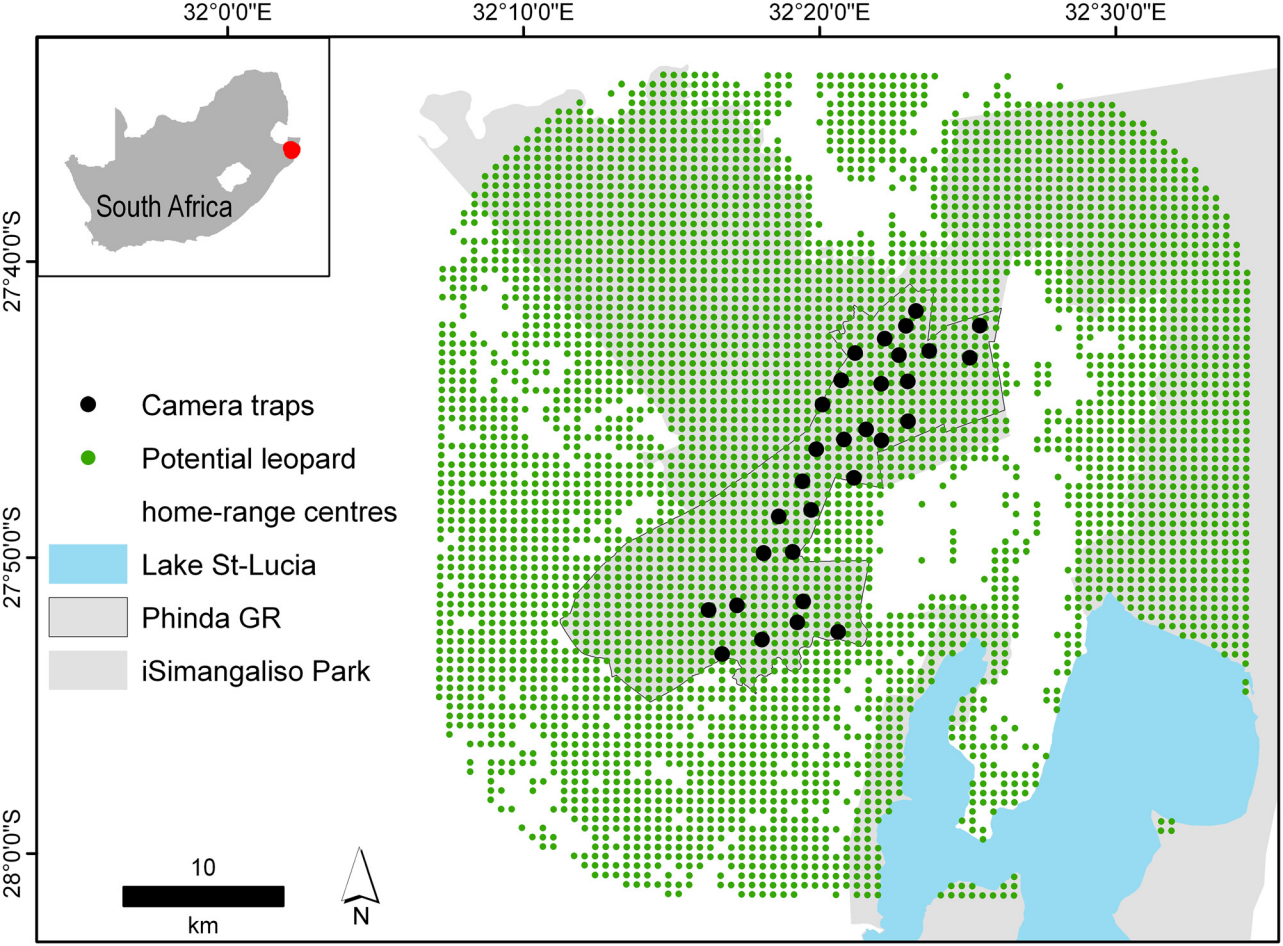
The location of the Phinda Private Game Reserve with camera-trap stations and potential leopard home-range centres (green). Patches of unsuitable leopard habitat are demarcated in white. Camera-traps were set on roads and trails to increase capture probability of leopards.

The study was conducted on Phinda Private Game Reserve (27° 51’ 30” S, 32° 19’ 00” E, hereafter Phinda) located in South Africa’s Kwazulu-Natal province, approximately 80 km south of the Mozambique border (Fig 1). Phinda (220 km^2^) is located adjacent to two large protected reserves, the Mkhuze Game Reserve and the St Lucia Wetland Park. The landscape is dominated by savanna interspersed with broad-leafed woodland, grassland and relict patches of Licuati Sand Forest, a threatened and fragmented tropical dry forest with high levels of plant and animal endemism. Further details on the study area and its faunal assemblage may be found in [27]. Importantly, Phinda also borders a number of private game reserves, cattle ranches and local communities. However, leopard hunting on the ranches surrounding Phinda has largely stopped allowing for a population recovery of leopards [9]. We therefore feel experimenting with lures during camera-trap surveys is warranted as leopards who have home-ranges beyond Phinda’s border are unlikely to encounter intentionally placed scent trails.

### Camera-trapping

We implemented two camera-trap surveys—a ‘control survey’ where no scent lures were deployed, which ran from 14 August–22 September 2012, and a ‘treatment survey’ where scent lures were deployed, which ran from 06 October–14 November 2012. Both surveys lasted 40 days and the camera-trap array remained consistent (Fig 1). We used Panthera^®^ IV digital camera-traps, set out in a paired format across 30 stations, totalling 60 camera-traps (1200 camera-trap nights). At least one camera-trap site was present per mean female leopard home-range (30 km^2^) [28] to ensure no animal had a zero probability of capture (mean camera spacing = 1.68 km) [5]. Camera-traps were mounted to wooden posts, 40 cm from the ground and were monitored every four days to replace memory cards and batteries, and to administer lures.

Leopards regularly use roads during territorial patrols and are often attracted to decomposing carcasses upon which they scavenge [29], [30]. Our lure therefore comprised a scent trail of decomposed entrails from the three main leopard prey species in Phinda, nyala *Tragelaphus angasii*, impala *Aepyceros melampus*, and warthog *Phacochoerus africanus* [27] that was deposited on the roads along which our camera-traps were placed. The majority of entrails were from nyala that had died during game translocation operations, which Phinda facilitated between June-November 2012. The other species were used if found opportunistically. The scent trail was laid on roads for a distance of 500 metres on either side of camera-traps and refreshed every four days. This protocol is similar to that employed by trophy hunters wishing to attract a leopard to a bait near a shooting hide [31]. However, as we provided no reward for leopards, we considered our scent trail a lure rather than bait. We created separate capture histories for leopards photographed in the control and treatment surveys, in order to compare closure estimates, distances moved by individuals, the times of their captures and density estimates. The identity of leopards was determined by the unique spot patterns on their pelage [32], and their sex was estimated using distinctive morphological features [33]. We used a Fischer’s exact test [34] to examine differences in capture rates among sex classes in the control and treatment surveys.

### Population closure

Leopards are long-lived (up to 19 years in the wild) [26]; hence, our survey period of 40 days seems sufficiently short to assume demographic closure [9]. We assessed geographic closure using the closure test of Otis et al [35] which assumes heterogeneity in recapture probability. We also used the closure test of Stanley and Burnham [36] which assumes a variation in time of recapture probability. Both tests were run in the statistical programme CloseTest version 3 (available online from: http://www.fort.usgs.gov/products/software/clostest/).

### Spatio-temporal patterning

We followed [8] in assessing the influence of lures on the spatio-temporal behaviour of leopards during the control and treatment surveys. We compared the maximum distance moved by individuals detected at more than one camera-trap during both surveys using a Wilcoxon Signed Ranks test [34]. We also examined the distribution of times when male and female leopards were captured on camera-traps during the two surveys. We sub-divided the photographic events into five distinct periods (00:00–06:00; 06:00–12:00; 12:00–16:00; 16:00–20:00; 20:00–00:00) for both males and females and compared their frequencies for both the passive and treatment surveys using a Fishers exact test.

### Density estimation

For comparison and consistency with previous non-spatial camera-trap surveys of leopards [5], [8], [21] we first used the software CAPTURE [35] to estimate leopard abundance. Following [5], we sub-divided our 40 sampling days into twenty 48-hour sampling occasions, and created a binary matrix of leopard detections, incorporating individual leopards only once during an occasion. CAPTURE provides the user with seven possible models for the computation of leopard abundance [35]. Each of these varies in its assumption regarding capture probability, which may be influenced by trap-specific response, time and individual heterogeneity [9]. CAPTURE computes a number of goodness-of-fit and between model statistics, and also has a discriminant function selection algorithm for objective model selection [35]. For each survey, we estimated the size of our effectively sampled area by adding a buffer equal to half the mean maximum distance moved by individuals photographed on more than one occasion (HMMDM) to our camera-trap grid [5] and removing zones of non-suitable leopard habitat (see supporting information). We divided the abundance estimate from CAPTURE by the effectively sampled area to estimate leopard density in the control and treatment surveys. We calculated variance for these density estimates using the delta method [10].

We also estimated leopard population density from both surveys using two spatially-explicit CR approaches (also using the 20 sampling occasion framework for comparability). Spatially-explicit CR estimates animal density from a set of individual animal detections made at capture locations nested within a broader network of potential leopard home-range centres [3], [6]. Through the incorporation of spatial information in the detection process the method is considered more robust to the “edge effects” common to non-spatial estimators and eliminates the need of a user-defined sampling area from which density is calculated [3]. We used the maximum likelihood based estimator secr version 2.9.3 [37] and the Bayesian estimator SPACECAP version 1.1 [6] in R version 2.15.2 [38]. Both methods make use of three input files: 1) detection history (information on animal identity, trap location and sampling occasion), 2) trap deployment (location of camera-traps, sampling occasions and camera function details) and 3) potential home-range centres file (a mesh of potential leopard home-range centres located in areas of suitable leopard habitat; these are demarcated by a 1 for suitable, and a 0 for non-suitable). We followed Gopalaswamy et al [6], and Athreya et al [39] in eliminating potential home-range centres from areas of non-leopard habitat. We used the raster [40] and rgdal [41] packages in R to create a rectangle around our outermost camera-traps. We then applied a 15-km buffer around a homogenous distribution of potential home-range centres spaced at 578 m intervals [6] and removed non-suitable leopard habitat (>3 settlements per 0.34 km^2^ grid and the St Lucia water body. A detailed habitat mask creation procedure is provided (S1 Protocol). Large felids are known to exhibit trap-specific and sex-specific traits in their ranging behaviours [9] and males are generally recorded at higher capture rates when compared to females [42], [43]. We therefore followed [19] and fitted models, in secr, which incorporated potential trap-specific responses of individuals and sex-specific detection probability (full model definitions created in secr may be found in Table 1). We maximised models in secr using conditional likelihood as this results in a likelihood, which ignores nuisance parameters within the model. We assessed candidate models in secr using AICc criteria [34] and evidence ratios (ER) [44]. In order to present leopard density, capture probability at home-range centre (g0) and sigma, we compared our top-ranked model with the next best-ranked model. We used the following formula:

ER=wj/wi

where *wj* is the AICc weight of our best-ranked model and *wi* is the AICc weight of the next best model (ie. Model being compared; Table 2). SPACECAP presently allows two models, 1) a base model which considers equal capture probabilities among individuals and 2) a model which incorporates a “trap-specific” response with detection probability increasing after initial capture in a trap [6]. Both models were fitted using a half-normal detection function and Bernoulli’s encounter model. The number of Markov-Chain Monte Carlo (MCMC) iterations and burn in varied per survey in order to achieve convergence (Table 3). SPACECAP uses data-augmentation, which adds to a dataset of known leopards with an enlarged set of all zero-encounter histories [6]. We set this augmentation value to 20 times the number of photographed individuals. We assessed model adequacy in SPACECAP through examination of the Bayesian *P-value* which is deduced from individual encounter frequencies. A Bayesian *P-value* close to either 0 or 1 is indicative of an inadequate model [6]. SPACECAP also provides a set of Geweke diagnostic statistics, which indicate whether MCMC chains have converged around a solution; if values range between -1.6 and 1.6 then convergence has been achieved [6]. We supplemented Geweke diagnostics supplied by SPACECAP by performing the Gelman-Rubin diagnostic and assessed potential shrink reduction factors for each parameter [45]. To do this we ran two more chains for each survey (including models incorporating the trap response variable), using different start values on the same data set (Table 4). Shrink Reduction Factors for each parameter should be <1.1 [45]. Finally we examined if estimates of density decreased using larger buffer spacings than the 15 km allocated in our models in secr using the mask.check function in secr, as too narrow a buffer is likely to produce inflated density estimates [46].

**Table 1:** Models used in secr analysis to estimate leopard density on Phinda.

Variable	Description	Variable function
g0~1	Constant	g0 and sigma kept constant
g0~b	Learned response	Step change in parameter after initial detection of animal
g0~h2	2-class mixture	Finite mixture model with two latent classes
g0~bk	Animal x site response	Site-specific step change
g0~Bk	Animal x site response	Site-specific transient response
g0~Sex*	Sex of animal	Male and female specific detection

**Table 2:** Model definitions and selection criteria for density models fitted for the control and lure surveys in secr.

Survey	Model definition	Parameters	Log likelihood	AIC	AICc	Delta AIC	AIC weight	Evidence ratio
	g0~bk	4	-213.34	434.67	438.67	0	0.97	1
	g0~1	3	-219.14	444.29	446.47	7.78	0.02	48.5
	g0~b	4	-218.21	444.42	448.42	9.75	0.01	97
Control	g0~sex	4	-218.59	445.18	449.18	10.5	0	-
	g0~h2	4	-218.59	445.18	449.18	10.5	0	-
	g0~Bk	4	-218.66	445.32	449.32	10.65	0	-
	g0~sex, sigma~sex	5	-217.47	444.94	444.94	12.93	0	-
	g0~sex, sigma~sex	5	-199.69	409.38	416.88	0	0.31	1
	g0~bk	4	-202.27	412.54	416.98	0.1	0.3	1.03
	g0~1	3	-205.21	416.42	418.82	1.94	0.12	2.58
Lure	g0~sex	4	-203.31	414.62	419.07	2.18	0.11	2.82
	g0~h2	4	-203.31	414.62	419.07	2.18	0.11	2.82
	g0~Bk	4	-204.56	417.13	421.57	4.69	0.03	10.33
	g0~b	4	-205.2	418.4	422.85	5.96	0.02	15.5

**Table 3:** Geweke diagnostic statistics and Bayes p-values generated from the four models run for the control and treatment surveys in SPACECAP.

Model	iterations	Burn in
Control survey trap absent	100 000	50 000
Control survey trap present	80 000	40 000
Treatment survey trap absent	80 000	30 000
Treatment survey trap present	80 000	40 000

**Table 4:** Shrink reduction factors generated using the Rubin-Gelman diagnostic in R.

Model	sigma		lam0		beta		psi		N	
	Point est	Upper C.I	Point est	Upper C.I	Point est	Upper C.I	Point est	Upper C.I	Point est	Upper C.I
Control survey trap absent	1.02	1.02	1.01	1.01	-	-	1	1	1	1
Control survey trap present	1.09	1.07	1.02	1.02	1.07	1.06	1.02	1.01	1.02	1.01
Treatment survey trap absent	1.01	1.01	1	1	-	-	1.01	1.01	1.01	1.01
Treatment survey trap present	1	1	1.07	1.05	1.16	1.12	1.01	1	1.01	1

## Results

### Photographic captures and population closure

Fifteen leopards (eight males, seven females) were captured on 40 occasions during the control survey, and fourteen leopards (10 males, four females) on 39 occasions in the treatment survey. Only one female with cubs was detected once during both surveys and those cubs were not considered in the analyses. Of the 15 leopards captured in the control survey, nine of these were present during the lure survey (six males, three females). We detected no significant difference in sex-specific captures between surveys (Fischer’s exact test, p = 0.90). The closure test of Otis et al [35] suggested no violation of permanent population closure for either the control (Z = 1.16, p = 0.88) or treatment (Z = 1.35, p = 0.91) surveys. Similarly, the test of Stanley and Burnham [36], which incorporates time variation in recapture probability, suggested population closure was achieved for both the control (X^2^ = 16.94, d.f = 14, p = 0.26) and treatment (X^2^ = 12.20, d.f = 15, p = 0.66) surveys.

### Spatio-temporal patterning

The maximum distance moved by leopards captured on more than one occasion was similar for the control (mean = 4.95 km, range = 0–11.8 km) and treatment (mean = 5.34 km, range = 0–10.4 km) surveys. Similarly, we found no difference in the maximum distance moved by specific individuals captured in both surveys (W = 31, p = 0.96). We found no difference in time-specific captures between the passive and treatment survey for males (Fisher’s Exact test, p = 0.79) and females (Fisher’s Exact test, p = 0.12).

### Photographic captures and density estimates

The heterogeneity model M(h) fit our data best for both the control and treatment surveys in CAPTURE. Using the jackknife estimator, CAPTURE estimated population abundance (SE) at 17±4.3 leopards for the control survey, yielding a density (SE) of 7.28±2 leopards/100 km^2^ when a buffer based on HMMDM (2.48 km) was applied to our survey area. Population abundance (SE) estimated by CAPTURE for the treatment survey was 23±6.6 leopards, resulting in a density (SE) of 9.28±2.9 leopards/100 km^2^ with a HMMDM buffer of 2.67 km.

From the seven models we created for the control survey in secr, there was very strong support for the trap-specific model, with the next best model being 48.5 times less likely. The trap-specific model estimated a density (SE) of 3.40±1.20 leopards/100 km^2^ (capture probability at home-range centre (g0 with SE) = 0.01±0.005; sigma (σ) = 4454.23±916.40 m). From the seven models created for our treatment survey, there was equal support for a model incorporating sex (as a function of both g0 and sigma) and the trap-specific model (ER 1 vs 1.03). The base model was also a candidate being 2.58 times less likely compared to the sex model. The sex model estimated a density (SE) of 3.47±1.22 leopards/100 km^2^; (capture probability at home-range centre for females (g0 with SE) = 0.03±0.01; sigma (σ) = 2541.03±674.90 m and (g0 with SE) = 0.03±0.01; sigma (σ) = 4366.64±969.99 m for males). The trap-specific model estimated a density (SE) of 3.28±1.27 leopards/100 km^2^ (capture probability at home-range centre (g0 with SE) = 0.02±0.007; sigma (σ) = 3843.36±935.87 m). Similarly, the base model from the treatment survey estimated a density (SE) of 3.30±1.15 leopards/100 km^2^ (g0 = 0.03±0.009; σ = 3414.81±712.49 m). The SPACECAP estimator yielded a density (SD) of 3.55±1.04 leopards/100 km^2^ (g0 = 0.03±0.01; σ = 3970±709 m) for the control survey in the absence of the trap response variable, and 3.65±1.22 leopards/100 km^2^ (g0 with SD = 0.01±0.001; σ = 4700±1010 m) using the trap response variable. The SPACECAP density (SD) estimate for the treatment survey was 3.43±1.12 leopards/100 km^2^ (g0 with SD = 0.03±0.01; σ = 3630±751 m) in the absence of the trap response variable, and 3.49±1.26 leopards/100 km^2^ (g0 with SD = 0.02±0.01; σ = 4170±1000 m) with the trap response variable. The 15 km buffer appeared to be adequate as density estimates remained unchanged with buffer increases at 22.38 and 28.84 km using the mask.check function in secr. Bayes p values and Geweke diagnostic statistics suggested model adequacy and convergence for all four models run in SPACECAP (Table 5). This was further confirmed by shrink reduction factors for key parameters which were all <1.1 (Table 4).

**Table 5:** Geweke diagnostic statistics and Bayes p-values generated from the four models run for the control and treatment surveys in SPACECAP.

Model	sigma	lam0	beta*	psi	N	Bayes p-value
Control survey trap absent	0.16	-1.15	-	-0.11	-0.11	0.53
Control survey trap present	-0.80	0.42	-0.25	1.01	1.09	0.53
Treatment survey trap absent	0.66	-0.36	-	-1.37	-1.17	0.59
Treatment survey trap present	0.26	0.31	0.81	0.37	-1.07	0.57

## Discussion

An important foundation for estimating population abundance and subsequent estimates of density using capture-recapture sampling is geographic closure [9]. Lures and other attractants may compromise geographic closure by prompting temporary immigration or emigration of animals into or out of a survey area. Although our results from the two closure tests suggest that there was no breach of geographic closure during the lure survey, this does not rule out a movement of individuals either inside or outside the camera-trap grid. We had no GPS collared leopards at the time of both surveys to confirm this. We however cannot discount the possibility that our closure tests suffered from a Type II statistical error. Gerber et al [8] suggested that the Pradel model [47] was more suitable for evaluating population closure (as it is highly flexible in modelling recapture variation and less susceptible to statistical errors when there is a trap-specific effect), but our sample sizes were inadequate to run this analysis. Importantly however, assumptions of geographic closure in SCR are more relaxed and are robust to temporary movements of individuals on the borders of the camera-trap grid [7].

The use of lures also did not appear to have a significant effect on the distances moved by leopards or the timing of leopard captures. Gerber et al [8] also found that the presence of a lure did not influence the distances moved by Malagasy civet *Fossa fossana* or the temporal distribution of their captures.

Importantly our model selection process revealed substantial evidence for the “site-specific learned response” model bk, with individuals becoming “trap happy” in the control survey. This result may seem surprising as the camera trap sites were not baited nor lured in the control survey. We would have rather expected trap shyness. The “site-specific learned response” also did not become more strongly positive when scent lures were used in the treatment. Instead there was an equal support for a model incorporating sex, the site specific behavioural model and the base model. The fact that we observed a behavioural effect when there was no lure, there is a possibility that there was some other unmodelled effect (possibly site-site detector differences). Additionally although the use of lures did not appear to affect the behaviour of leopards (behaviour being defined as the distances they moved between camera-traps and when they were caught on camera-traps), they also did not improve capture or recapture rates, and hence the precision of our density estimates. This may be due to the limited range over which our lures were effective. Felids do not possess a particularly acute sense of smell [48]; hence, leopards were only likely to detect scent trails in close proximity. It is also possible that leopards may be more predisposed to scavenging, and thus to respond to lures, in more arid areas where prey abundance is lower. Phinda is a productive system and earlier research demonstrated that the leopard population was not constrained by prey availability [49].

Studies on leopards elsewhere and carnivores more broadly suggest that capture rates may be increased by using baits (i.e. where the target species is rewarded) rather than lures. Grant [21] placed stillborn cattle foetuses near camera-traps in Mangwe, Zimbabwe, and showed a twenty-fold increase in the number of leopard captures when compared to a failed non-baited survey. Similarly, a study in the Bubye valley conservancy [19] found that the presence of baits near camera-traps increased leopard captures four-fold. Although at the outset these results appear promising, the precision estimates of Du Preez et al. [19] only increased marginally with the presence of baits (2–4%) and at an additional cost of 314 man-hours per survey [50]. Leopards can remain sedentary, feeding on a single carcass for up to a week if it is large enough [29]. Such a change in an individual’s daily routine may be significant in the context of a 40-day camera-trap survey, especially if non-spatial methods are used. Specifically in reducing the number of detections of an individual leopard on different camera-traps, which could underestimate the movement parameter sigma. However SCR models enable the user to adjust the encounter probability, sigma and density when a trap-specific response is added to the model. Even when using non-spatial HMMDM [19] suggested no significant changes in the ranging behaviours of leopards in their study, although they did not explicitly test for closure violations. The detection distance of baits may also be larger than lures, artificially drawing individuals into the sampling area and potentially biasing population estimates.

The density estimates from the two spatially-explicit capture-recapture approaches (maximum likelihood and Bayesian) were comparable, but were significantly lower than estimates derived using traditional, non-spatial methods. This concurs with previous research which shows that non-spatial CR analyses typically overestimate population density by underestimating the distances moved by animals [51], [52], [53]. Furthermore, spatially-explicit CR models are generally more robust to changes in the camera-trap array, the sizes of sampled areas and the movement patterns of individuals [54]. This was reflected in our study by a 22% increase in density estimates between the control and treatment surveys using the non-spatial estimator, compared to a nominal change using spatial estimators. It was highly unlikely that leopard density varied much over our study given its duration and the short interval between surveys. This was reflected by the similar density estimates obtained from our candidate models in secr and SPACECAP over the combined survey period.

The application of scent trails was laborious and difficult to justify for our study given the lack of improvement in the precision of density estimates. However, capture probabilities at home-range centre among our surveys were similar to other recent studies employing spatially-explicit CR methods to estimate leopard density (eg. in the Limpopo province of South Africa [46], p = 0.03; and a post conflict landscape of Cambodia [55], p = <0.01–0.04), and were sufficiently high to produce reliable population estimates even in the absence of attractants. This may not be the case in lower density populations, or for species, which are less routine in their movement patterns and thus more difficult to camera trap. In such cases the use of an attractant may be warranted. For felids, this may require the deployment of baits rather than lures; however, we strongly recommend that this only be done after the effects of baiting on population and spatial parameters have been assessed, preferably using a similar approach as this study (i.e. with a control and treatment period). Lures may be adequate for species with superior olfactory senses such as hyaenids [16] or viverrids [8], where they are likely preferable to baits. The use of lures could potentially also be warranted if a certain variety is found to be particularly successful. Thorn et al [16] and Braczkowski et al [18] followed this approach in the Limpopo and Cape respectively, and attempted to examine lure preference.

An alternative way to increase the capture rates of animals during surveys (and consequently measures of precision) is by lengthening the duration of surveys. This is indeed recommended by Tobler et al [2], who suggest that higher captures be preferred over short survey periods. Borchers et al [56] also developed a continuous time SCR model and showed that using count (rather than binary) data improved parameter estimates. Both of these approaches could help to increase the precision of density estimates.

Our results suggest that although using lures do not lead to the violation of CR model assumptions in a savanna environment, they also do not improve the precision of density estimates, so may be of limited use for leopard researchers. Insights such as these are important in assisting biologists and conservation managers in determining which approaches should be applied or avoided for the most cost- and time-efficient method to enumerate leopards and potentially other large carnivores.

## Supporting Information

S1 Dataset SPACECAP control survey captures. (CSV)

S2 Dataset SPACECAP lure survey captures. (CSV)

S3 Dataset SPACECAP trap layout. (CSV)

S4 Dataset SPACECAP habitat suitability mask. (CSV)

S5 Dataset secr control survey captures. (TXT)

S6 Dataset secr lure survey captures. (TXT)

S7 Dataset secr survey trap layout. (TXT)

S1 Protocol Suitable leopard habitat mask creation procedure implemented in the R statistical environment. (DOCX)

S1 Results SPACECAP control survey trap absent output. (TXT)

S2 Results SPACECAP control survey trap present output. (TXT)

S3 Results SPACECAP lure survey trap absent output. (TXT)

S4 Results SPACECAP lure survey trap present output. (TXT)
