# Supplementary material for: Scent Lure Effect on Camera-Trap Based Leopard Density Estimates
Source: PLoS One. 2016 Apr 6;11(4):e0151033. doi: 10.1371/journal.pone.0151033 (PMC4822812; doi:10.1371/journal.pone.0151033)
Supplement: S1 Protocol — (DOCX) [file pone.0151033.s008.docx]

**Supporting information S1. Habitat suitability mask protocol**

Both “secr” and “SPACECAP” require the user to create a habitat suitability mask which denotes areas in the landscape which are likely to feature potential leopard home-range centres [6], [34]. In order to do this we used the rgdal package in R to create a polygon around our outermost camera traps and buffered it by 15 km [6]. This buffer width has been suggested for large carnivores such as tigers *Panthera tigris*, pumas *Puma concolor* and jaguars *Panthera onca* [6] and is applied to ensure that animals residing outside it are not detected by camera traps within the buffered area. We then followed the example of Chapman (2012) and Whittington-Jones (2011) in identifying areas of potential leopard home-range centres. We overlaid human settlement density data (Electricity supply commission, South Africa) over 0.336 km^2^ grids in our 15 km buffer (the recommended range centre spacing; [6]) and removed centres located within a grid with ≥ four settlements outside of protected reserves (Figure 1; SI). Leopards regularly frequent lodges and rest camps in the protected Mkhuze and Phinda reserves and even bear cubs there (Balme unpublished data; [25]), we therefore considered these settlement varieties as locations of potential range centres. We believe the approach using human settlements is a prudent one, particularly in the broader landscape outside of Phinda as historic leopard surveys on cattle farms in the region, suggest leopard presence follows a marked decline along the gradient of reserve protection from Mkhuze, Phinda and finally cattle/pineapple farms [8].
